# Diabetic Uremic Syndrome Presenting Reversible Parkinsonism with Bilateral Basal Ganglia Lesions: A Case Report

**DOI:** 10.31662/jmaj.2021-0101

**Published:** 2021-12-03

**Authors:** Tomohiro Suzuki, Syuichi Tetsuka, Tomoko Ogawa, Ritsuo Hashimoto, Hiroyuki Kato

**Affiliations:** 1 Department of Neurology, International University of Health and Welfare Hospital, Nasushiobara, Japan

**Keywords:** basal ganglia lesion, diabetic uremic syndrome, dopamine transporter, lentiform fork sign, parkinsonism

## Abstract

The patient was a 57-year-old man with a 15-year history of diabetes mellitus and a 3-year history of dialysis. He developed a subacute onset of Parkinsonism, including gait disturbance, bradykinesia, cogwheel rigidity, and myoclonus attacks. Magnetic resonance imaging (MRI) of the brain revealed swollen bilateral basal ganglia lesions, which appeared hyperintense with the lentiform fork sign on fluid-attenuated inversion recovery images, indicating vasogenic edematous lesions. He was diagnosed with diabetic uremic syndrome. Dopamine transporter single-photon emission computed tomography revealed no decrease in dopamine transporters. After approximately 4 weeks of continuous hemodialysis, rehabilitation, and supportive therapy, his neurological symptoms and MRI findings markedly improved. Although this disease has been reported in a few cases, its etiology and treatment remain unclear. In this case of diabetic uremic syndrome, dopamine secretion capacity was normal even though the patient had parkinsonian symptoms. This finding might contribute to further elucidation of the pathological mechanism of diabetic uremic syndrome.

## Introduction

Diabetic uremic syndrome is a disease concept that was proposed by Wang et al. in 2003 ^(1)^, which is characterized by the presence of bilateral basal ganglia lesions, acute to subacute Parkinsonism, impaired consciousness, involuntary movements, and reversible imaging findings and symptoms ^(2), (3)^. Herein, we present a case of chronic maintenance hemodialysis with a similar course.

## Case Report

The patient was a 57-year-old man who was diagnosed with diabetes since age 42 but had not received any treatment. At age 53, he was diagnosed with diabetes-induced renal failure and retinopathy, and his renal function was gradually deteriorating. At age 54, maintenance hemodialysis was started. He was admitted to our hospital because he began experiencing difficulty in walking, approximately 1 week prior. On neurological examination, he was generally conscious. He had poor facial expressions and a guttural voice. It took time for him to respond, indicating bradyphrenia. The patient demonstrated slow movements, postural myoclonus in the upper and lower limbs, and significant right-sided cogwheel rigidity in the upper and lower limbs, but no resting tremor was observed. He was unable to maintain a sitting position and had asterixis. There was a mild abnormality in his motor coordination, manifested by mild dysmetria and decomposition in the Finger-to-Nose-Test, and he was unable to stand or walk due to truncal ataxia. The deep tendon reflexes in both legs were normal, and the Babinski and Chaddock reflexes were negative. Vibration sensation in both lower limbs decreased mildly.

**Figure 1.**
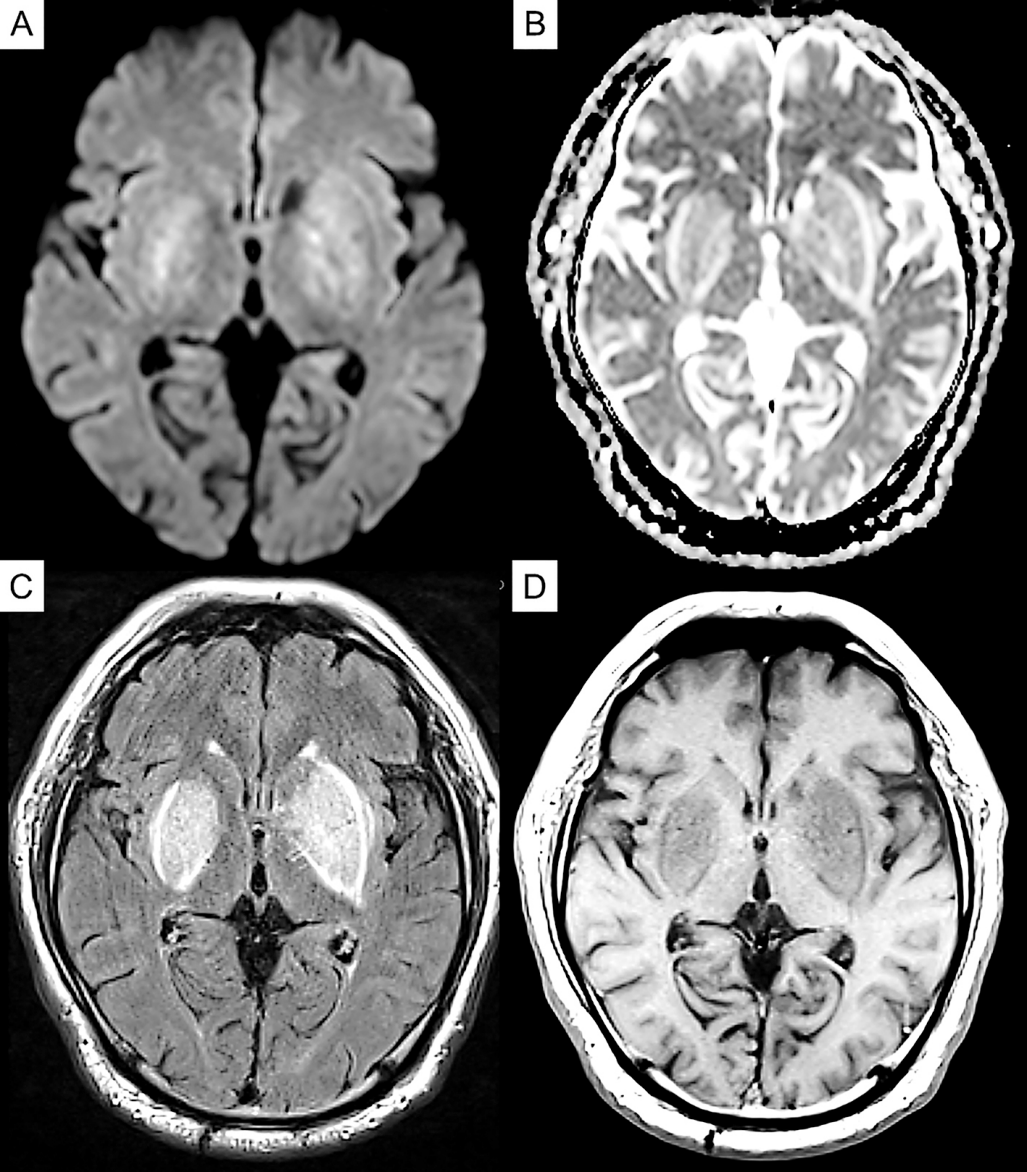
Axial brain magnetic resonance imaging (MRI) on admission. (A) Diffusion-weighted image revealing mild symmetrical restricted diffusion over both basal ganglia. (B) The lesion was hyperintense on the apparent diffusion coefficient map. (C) Fluid-attenuated inversion recovery image showing hyperintensity in the lentiform nuclei and head of the caudate nuclei with surrounding edema in the internal, external, and extreme capsule regions. (D) T1-weighted magnetic resonance image demonstrating slight low-signal changes.

**Figure 2.**
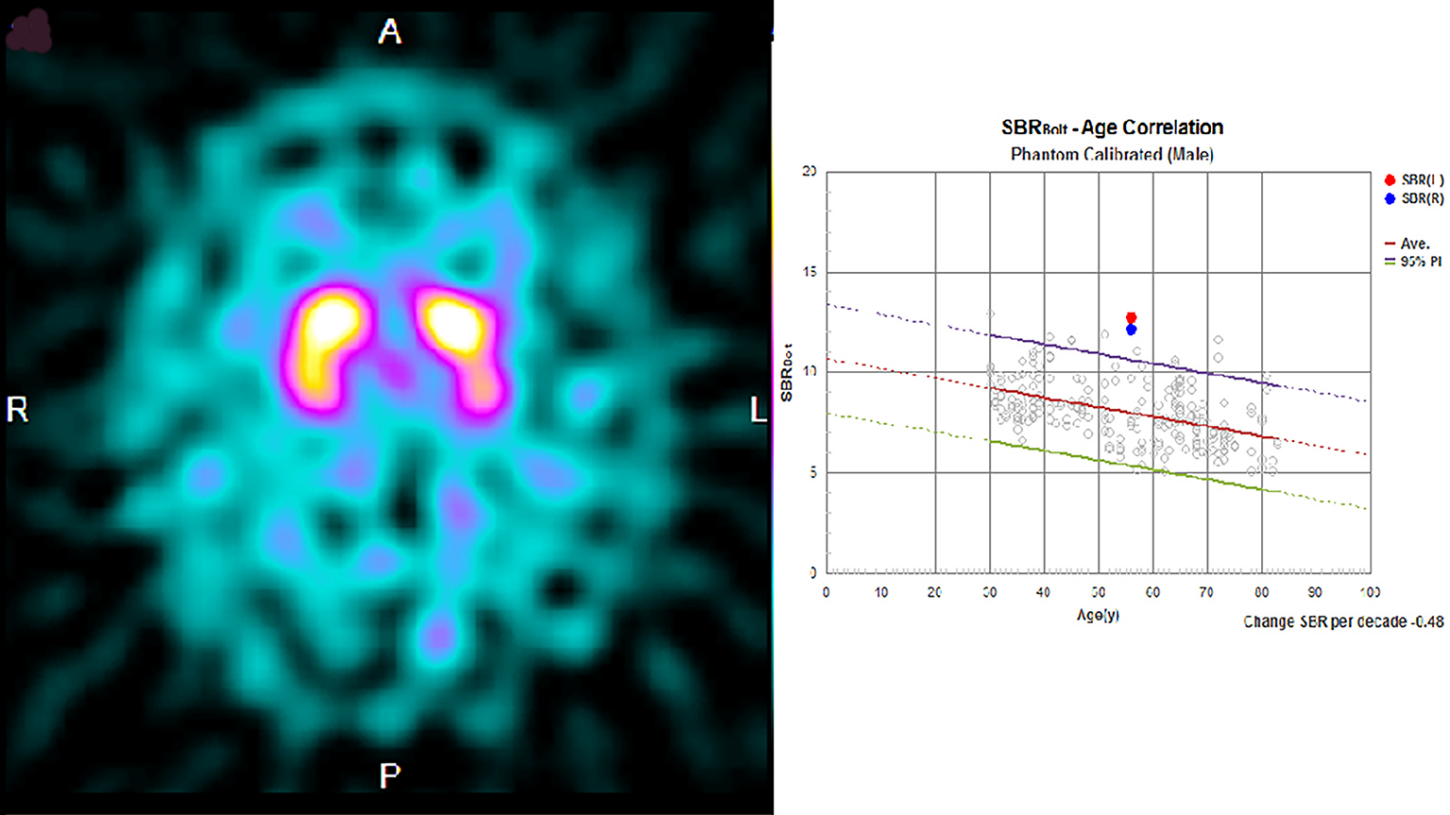
Dopamine transporter single-photon emission computed tomography imaging using ioflupane I 123 injections. Normal finding of the patient diagnosed with diabetic uremic syndrome.

Laboratory exploration on admission revealed renal dysfunction (blood urea nitrogen, 8.8 mg/dl; creatinine, 8.37 mg/dl; sodium, 138 mEq/dl; potassium, 3.4 mEq/dl; calcium, 9.6 mg/dl; phosphorus, 2.4 mg/dl). As for glycemic control, blood glucose was 145 mg/dl, and hemoglobin A1c was 6.0%. His white blood cell count and C-reactive protein concentration were 6,710/μl and 0.82 mg/L, respectively. Serum antinuclear antibody, anti-Ro/SSA, anti-La/SSB antibody, and anti-neutrophil cytoplasmic antibodies targeting myeloperoxidase and proteinase 3 were negative, and the serum immunoglobulin (IgG, IgM, and IgA) and blood ammonia levels (42 μg/dL) were normal. Cerebrospinal fluid examination revealed an increase in protein levels (117 mg/dl), but pleocytosis was not observed. Magnetic resonance imaging (MRI) of the brain revealed symmetrical hyperintensity with tumefactions in both capsules, globus pallidus, and external capsule, with a bright hyperintense rim delineating the lentiform nucleus on fluid-attenuated inversion recovery images, which is known as the “lentiform fork sign.” T1-weighted images revealed low-intensity signals in the same areas. Diffusion-weighted imaging (DWI) revealed a faint high signal, whereas the apparent diffusion coefficient (ADC) revealed a high signal (Figure 1). Therefore, DWI findings were considered T2 shine-through, reflecting vasogenic edema. Dopamine transporter single-photon emission computerized tomography (SPECT) using ioflupane I 123 injections was conducted to check for the presence of Parkinson’s syndrome, and there was no decrease in dopamine transporters (Figure 2).

**Figure 3.**
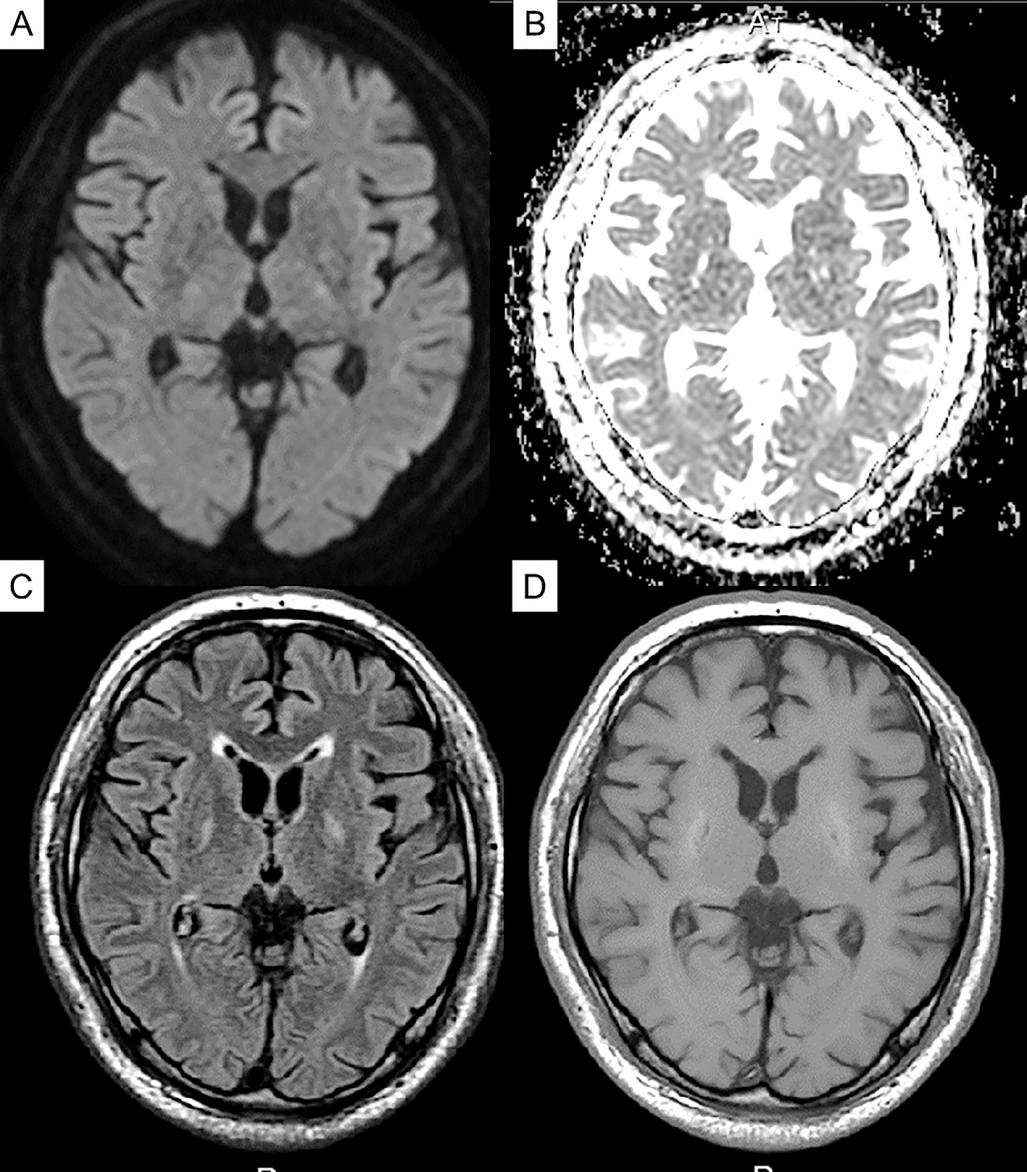
Follow-up magnetic resonance imaging (MRI) obtained on day 30 after admission. (A) Diffusion-weighted image. (B) Apparent diffusion coefficient (ADC) map. (C) Fluid-attenuated inversion recovery (FLAIR) image. (D) T1-weighted image. FLAIR revealing a slightly high signal in both basal ganglia, which were significantly weaker than the initial MRI signal. FLAIR image and ADC map revealing a slit-like high intensity in the globus pallidus; the intensity of these same areas was decreased on T1-weighted images.

The patient was diagnosed with diabetic uremic syndrome based on clinical and imaging findings, and hemodialysis was continued while rehabilitation was commenced. After admission to our hospital, the patient tended to be somnolent and required assistance in sitting up on day 1, but awakening improved on day 7, and he was able to hold a standing position and walk in a circle on day 14. On day 20, he was able to walk alone. In addition, myoclonus in his limbs was no longer apparent. Brain MRI also revealed an improvement in the abnormal signal area with the clinical course (Figure 3). On day 13, symptoms such as sluggish movement and gait disturbances seen on admission had nearly disappeared.

## Discussion

We reported the case of a patient with diabetes on hemodialysis who had subacute reversible Parkinsonism and presented with the lentiform fork sign on brain MRI ^(4)^. Based on the MRI, DWI, and ADC values in the acute phase of the disease, previous evaluations revealed that the basal ganglia lesions present with mixed findings of vasogenic and cellular edema ^(5), (6)^. In the chronic stage, the high DWI signal disappears; however, the high signal and increased ADC value on the slit-like T2-weighted image of the globus pallidus remains, which is characterized as irreversible cystic degeneration ^(6)^, and the time course of MRI in this case also revealed similar findings. Dopamine transporter SPECT revealed no significant bilateral decrease in dopamine transporter, which indicated the lack of degeneration and dropout of dopaminergic neurons in diabetic uremic syndrome. To the best of our knowledge, this is the first case report to show that dopaminergic neurons do not degenerate or drop out in diabetic uremic syndrome, despite the presence of symptoms of Parkinson’s disease. Ishii et al. reported that (^11^C)-labeled 2-carbomethoxy-3-(4-fluorophenyl) tropane and (^11^C)-labeled raclopride revealed significant bilateral reductions in pre- and postsynaptic functions of the dopaminergic neurons in a patient with diabetic uremic syndrome ^(7)^. However, in their case, the parkinsonian symptoms were irreversible, and myocardial scintigraphy using meta-iodobenzylguanidine revealed a reduction of the heart/mediastinum ratio, which might indicate the possibility that their patient developed Parkinson’s disease because of uremic encephalopathy.

Based on the results of the dopamine transporter SPECT, postsynaptic dysfunction was presumed to be the main pathogenesis of the Parkinsonism observed in this patient, and we personally think that it is better to consider that there was functional impairment during the acute phase. Although the pathogenesis of this disease is unclear, vasogenic edematous changes may occur in the basal ganglia, and diabetic uremic syndrome develops because the basal ganglia are particularly susceptible to toxic substances and metabolites in the brain ^(8)^. However, it is impossible to conclude from MRI findings alone that the Parkinsonism observed in this case does not involve organic damage to the substantia nigra-striatal pathway. Here, the results of the dopamine transporter SPECT suggested that the substantia nigra-striatum system was preserved; therefore, we did not think L-dopa treatment would be effective in this patient; hence, it was not given. We believe that the results of the dopamine transporter SPECT were useful in inferring the pathology of this condition. The demonstration of normal dopamine secretion in diabetic uremic syndrome may contribute to further elucidation of the pathological mechanism.

## Article Information

### Conflicts of Interest

None

### Author Contributions

All authors revised the manuscript, approved the manuscript to be published, and agree to be accountable for all aspects of the work in ensuring that questions related to the accuracy or integrity of any part of the work are appropriately investigated and resolved.

### Informed Consent

Written informed consent for the publication of this case report was obtained from the patient and his family.
